# Environmental exposures and lymphoma risk: a nested case–control study using the Golden Retriever Lifetime Study cohort

**DOI:** 10.1186/s40575-022-00122-9

**Published:** 2022-07-15

**Authors:** Kristofer R. Luethcke, Lauren A. Trepanier, Ashleigh N. Tindle, Julia D. Labadie

**Affiliations:** 1 University of Wisconsin-Madison School of Veterinary Medicine, Madison, WI USA; 2 Scientific Programs Department, Morris Animal Foundation, Denver, CO USA

**Keywords:** Canine lymphoma, Epidemiology, Air pollution, Environmental exposures, Secondhand smoke, Cancer risk

## Abstract

Lymphoma is the second most common cancer affecting Golden Retrievers and is hypothesized to arise through a complex interaction of genetic and environmental factors. The aim of this nested case–control study was to investigate the association between potential environmental pollutant sources and lymphoma risk among Golden Retrievers participating in the Golden Retriever Lifetime Study. Forty-nine Golden Retrievers with non-cutaneous lymphoma and 98 Golden Retrievers without a history of cancer matched by age, sex and neuter status were selected from the Golden Retriever Lifetime Study cohort. Geographic proximity between each dog’s primary residence and nine potential sources of environmental pollution was determined. In addition, the average annual ozone and airborne fine particulate matter levels for each dog’s county of residence and owner-reported secondhand smoke exposure were evaluated. Environmental pollution sources of interest included chemical plants, municipal dumps, manufacturing plants, incineration plants, railroad embankment tracks, landfills, coal plants, high-voltage transmission lines, and nuclear power plants. Conditional logistic regression was used to estimate odds ratios (ORs) and 95% confidence intervals (CIs) for each exposure of interest. Subgroup analyses were conducted to evaluate whether associations differed among 1) dogs with multicentric lymphoma, 2) dogs with B-cell lymphoma, and 3) dogs with T-cell lymphoma. No variables reached statistical significance when evaluating all cases together. However, cumulative exposure burden (household proximity to 3 or more pollution sources) approached significance within the multicentric lymphoma subgroup (OR = 2.60, 95%CI 0.99–6.86, *p*-value = 0.053). Patterns emerged among B- and T-cell subgroups, but none reached statistical significance. Ongoing research is warranted to discern if different environmental mechanisms may be driving B- and T-cell lymphoma immunophenotypes, consistent with previously reported regional differences in subtype prevalence.

**Supplementary Information:**

The online version contains supplementary material available at 10.1186/s40575-022-00122-9.

## Plain English Summary

Lymphoma is a common cancer affecting dogs, particularly Golden Retrievers. By identifying risk factors for lymphoma, work can be done to reduce harmful exposures or increase monitoring among dogs at a higher risk of disease. Using a subset of dogs from the Golden Retriever Lifetime Study, we sought to investigate whether dogs with lymphoma were more likely to live near certain environmental pollutant sources than dogs without lymphoma.

Forty-nine Golden Retrievers with non-cutaneous lymphoma and 98 Golden Retrievers without a history of cancer were selected from the Golden Retriever Lifetime Study Cohort. We evaluated how close each dog lived to nine environmental pollutant sources: chemical plants, municipal dumps, manufacturing plants, incineration plants, railroad embankment tracks, landfills, coal plants, high-voltage transmission lines, and nuclear power plants. Additionally, we evaluated individual exposure to secondhand smoke, and average annual ozone and particulate matter exposure (as surrogate measures for air pollution) for each dog’s county of residence.

None of the exposures examined were associated with an increased lymphoma risk in this population. More research is needed, including direct biomonitoring, to determine whether specific environmental exposures are associated with lymphoma in the Golden Retriever breed.

**Supplementary Information:**

The online version contains supplementary material available at 10.1186/s40575-022-00122-9.

## Introduction

Lymphoma, a heterogenous malignancy of the lymphatic system, is one of the most common cancers in dogs [1]. The etiology of lymphoma is complex, involving both genetic and environmental factors. Certain breeds, including Golden Retrievers, Boxers, Bulldogs, Doberman Pinschers, Rottweilers, Bernese Mountain Dogs and German Shepherd Dogs, are overrepresented, suggesting a genetic risk component [2, 3]. However, studies have identified geographic differences in lymphoma prevalence and subtype distribution, even among high-risk breeds, indicating environmental influences in disease risk [3–7] Certain chemical exposures have previously been associated with lymphoma risk in dogs, including commercially applied pesticides, commercially and individually applied herbicides (especially 2,4- dichlorophenoxyacetic acid) [4, 8, 9], individually applied insect growth regulators [9], and individual household chemical use of paints and certain solvents [10]. Environmental exposures including proximity to industrial areas, waste incinerators, polluted sites and radioactive waste have also been implicated [10, 11], as well as proximity to nuclear power plants, chemical suppliers, or crematoriums [6]. Secondhand smoke exposure has also been associated with lymphoma risk in dogs [12].

Studies in human non-Hodgkin’s lymphoma (NHL), which closely approximates canine lymphoma in morphology and biological activity [13], offer additional insight into potential risk factors for lymphoma in dogs. The geographic distribution between human and canine lymphoma cases is correlated, with an increased prevalence for both species associated with residing in industrial areas [6, 10, 11]. These parallel geographic patterns of lymphoma risk among dogs and people suggest shared etiologic mechanisms and environmental risk factors. Studies have further characterized increased lymphoma risk in people with exposure to specific pollutant compounds, including aromatic hydrocarbons such as benzene from exhaust, secondhand smoke, and petrochemical solvents [7]. Volatile organic compounds, as found in paint remover, cleaning solvents, industrial laminates, rubber, plastic, insulation material and fiberglass, are also associated with increased lymphoma risk in humans [14, 15]. As with dogs, environmental treatments including pesticides [16, 17], herbicides, and fungicides [18] are associated with increased lymphoma risk in people, especially among those employed in commercial application fields. In one study, proximity to high-voltage electrical lines was associated with lymphoma risk in humans, especially during early-age exposure, among residents of Tasmania [19]. Many of the above listed risk factors correlate with degree of urbanization. Ozone and airborne particulate matter (2.5 microns or less, PM_2.5_) have been used as measures of overall air pollution burden and level of industrialization in a region, each of which has correlated with risk of a variety of diseases, including cancer, in humans and dogs [6, 20–24]. In addition, tobacco smoke contributes to indoor air pollution and is a risk factor for certain types of NHL in people [25].

While Golden Retrievers are overrepresented among dogs with lymphoma in the United States [2, 26], it is not yet fully understood why some develop lymphoma and others do not. Current literature identifies increased risk among dogs and people with certain environmental exposures, especially those centering around urbanicity and industrial pollution. We therefore hypothesized that these exposures might also influence lymphoma risk among Golden Retrievers. In this study**,** we aimed to assess the association between lymphoma risk and residential proximity to local environmental pollution sources, indicators of air quality, including average annual ozone and PM_2.5_ levels by county of residence, and owner-reported secondhand smoke exposure using a nested case–control study from the Golden Retriever Lifetime Study (GRLS). As a secondary goal, we evaluated subtype-specific lymphoma risk. The findings from this study are intended to help direct future studies on canine and human lymphoma risk monitoring.

## Methods

### Study design

This study was conducted in conjunction with the Morris Animal Foundation GRLS cohort. Details about the GRLS participant recruitment and data acquisition have been previously described [27]. Briefly, privately owned pedigree-confirmed Golden Retriever dogs between the age of 6 months to 2 years old were recruited from across the continental United States between the years of 2012–2015, for a longitudinal lifetime study. All participating owners and veterinarians gave informed consent and agreed to complete all requirements of the study prior to enrollment. Data from each participating dog includes a complete annual veterinary physical examination, banked relevant biological samples (whole blood, DNA, serum, urine, feces, hair, and toenails), internet-based questionnaires about the dog completed by the owner and veterinarian, and any diagnostic, pathology or procedural reports at the time of diagnosis of a malignancy. Study methods and participation requirements were reviewed and approved by the Morris Animal Foundation’s appointed Animal Welfare Advisory Board.

For the purposes of our study, a nested matched case–control design was utilized to investigate the association between household proximity to environmental pollutant sources, county annual average ozone and PM_2.5_ levels, secondhand smoke exposure, and lymphoma risk in Golden Retrievers.

### Selection criteria

Fifty cases of non-cutaneous lymphoma were identified prior to January 1, 2021, diagnosed via histopathology, cytology, flow cytometry, or polymerase chain reaction for antigen receptor rearrangement (PARR). Immunophenotyping data and additional subtyping were recorded when available and dogs were classified as B-cell or T-cell lymphoma when possible. Cases with in-house cytology only (*n* = 1) were excluded. Age at definitive diagnosis, sex, and age at gonadectomy (if performed) were obtained from annual veterinarian questionnaires.

Control dogs identified were free from diagnosis of any neoplastic disease and were matched to each case by sex, age at gonadectomy (within 3 months of case gonadectomy age, if applicable), timing of the annual veterinary visits (within 6 months of matched case annual visit exams), and age at the veterinary visit within 6 months to the case age at diagnosis. Two controls were matched to each case.

### Household of longest residence

For each dog, household proximity to pollutant sources of compounds previously associated with lymphoma risk in humans or dogs was determined, as well as the Environmental Protection Agency (EPA)-generated average annual 4^th^ max 8-h average ozone (in ppm) and average annual PM_2.5_ concentration for each dog’s county of longest residence. The home address (and county) for each dog was defined as the address at which the case or control dog has lived for the longest duration while enrolled in GRLS, up to the date of diagnosis (for case dogs) or age-match (for control dogs). If any of the annual questionnaires had a missing address for a study year, the dog was assumed to have been living at the same address as the year immediately prior. If the dog moved multiple times from the start of enrollment to date of diagnosis or match, and the dog was at two or more addresses for an equal amount of time, the most recent of the addresses in the tie was used. The mobility of the cohort was analyzed through approximate years lived at the residence of longest duration, and number of times the dog moved up to the date of diagnosis or case-match. The time lived at the longest residence was approximated based on the number of study years the dog was reported to live at that home. Therefore, if a dog moved halfway through a study year, they were counted as having lived a full year at that new residence as of the year of the annual questionnaire on which the new address was reported.

### Geocoding household proximity to environmental pollutant sources

The geocoding method used for this study has been previously described [28]. Briefly, for each address, the Google Maps “nearby” function was used in conjunction with a-priori search terms (Table 1) to determine a binary (yes or no) response for household proximity to environmental pollutant sources. A two-mile radius was used for chemical plants, municipal dump, landfills, rubber, leather or textile manufacturing plants, coal plants, active incinerators, and railroad embankment tracks. This was based on a predicted radius in which dogs might be walked outside. A 50-m radius was used for high-voltage transmission lines (based on a prior study of childhood leukemia risk among residents of Tasmania [19]), and a 10-mile radius was used for nuclear power plants, based on previous findings in Boxers with lymphoma [6]. Each site was verified through a combination of physical map inspection and source website data to confirm the site was active and a primary source of associated pollutants (for example, confirming an in-house crematorium for a given funeral home location, and differentiating between distributing plants, corporate offices, and genuine manufacturing plants for manufacturing companies). Sources that appeared under more than one search term (e.g., a landfill site appearing both under the landfill search and the waste management search) were only counted once. Google Maps data are updated at least every 2 years. Most cases and controls recruited for this study were residing in their address of longest residence at the time geocoding was conducted, and the majority had resided there for at least 2 years. Thus, an assumption was made that the geographic data presented on Google Maps was representative of the relevant living environment for each dog.

**Table 1:** Pollutant sources, search terms, and search distance used to determine household proximity to potential carcinogens

Pollutant source	A priori “nearby” search terms	Mile-radius from household
Chemical plant	Chemical plant, chemical manufacturer	2
Municipal dump	Municipal dump, city dump, garbage dump, dump site, waste management	2
Landfill	Landfill	2
Manufacturing plant	Rubber/leather/textile manufacturer, rubber/leather/textile plant, textile mill	2
Coal plant	Coal plant, coal power plant, coal power station	2
Incineration plant	Incinerator, incineration plant, crematorium, cremation, funeral	2
High-voltage power line	None: physical Google Earth search for high voltage lattice tower within search radius	50 m (200 ft)
Railroad embankment tracks	None: physical Google Earth search for embankment tracks within search radius	2
Nuclear power plant	Nuclear reactor, nuclear power plant, nuclear power station	10

An overall exposure score was also created summing the binary responses for nine individual environmental pollutant sources as a proxy for overall pollutant burden. This was evaluated as a categorical variable split at the midpoint score (< 3/ ≥ 3).

### Residential county ozone data

EPA-generated annual 4^th^ max 8-h average ozone (ppm) and weight annual mean PM_2.5_ concentrations (ug/m^3^) for each dog’s county of longest residence was acquired from publicly available data on the EPA website between the years of 2013–2019. For each county, the average 4^th^ max 8-h average ozone over the years of residence was calculated and subsequently dichotomized based on the EPA regulatory limit of 70 ppb. The concentration of PM_2.5_ over the years of residence was treated as a continuous variable due to a low number of residences exceeding the EPA regulatory limit of 12 ug/m^3^.

### Secondhand smoke data

Owner-reported data on secondhand smoke exposure were obtained through the GRLS Annual Owner Questionnaire. Owners were asked to report “the average number of hours per day, over the past 12 months, your dog has been exposed to secondhand smoke (from all sources including, cigarettes, cigars, pipes).” Responses were averaged across all Annual Owner Questionaries up to the time of diagnosis or match. Values were subsequently dichotomized as any versus no secondhand smoke exposure.

### Statistical analyses

Variables were summarized as frequency and percent or median and range as appropriate. Univariable conditional logistic regression was used to assess the relationship between lymphoma risk and 1) household proximity to each pollutant source of interest, 2) cumulative exposure score of ≥ 3 pollutant sources (yes/no), 3) average annual county-level EPA ozone level ≥ 70 (yes/no), 4) average annual county-level PM_2.5_ levels, and 5) any secondhand smoke exposure during the study (yes/no). Odds ratios (ORs) and 95% confidence intervals (CIs) were calculated using R version 4.0.3.

Exploratory secondary analyses were performed using 1) only cases of multicentric lymphoma, 2) only cases with B-cell immunophenotype, and 3) only cases with T-cell immunophenotype to decrease heterogeneity and determine whether proximity to pollutants may differentially associate with lymphoma subtype-specific risk.

## Results

### Study demographics

Forty-nine cases and ninety-eight controls met our inclusion criteria. Most dogs (67%) were spayed or neutered, with relatively even numbers of males (53%) and females (Table 2). The median age of lymphoma diagnosis was 5.8 years (range 1.7–9.6). Dogs were relatively evenly divided across five US geographic regions. Most dogs (80% of cases, 76% of controls) did not move their primary residency between the date of enrollment and date of diagnosis or match; the median duration of stay at their longest residence was 5–6 years.

**Table 2:** Study population demographics, stratified by lymphoma status

		**Case**	**(*n* = 49)**	**Control**	**(*n* = 98)**
		n	(%)	n	(%)
**Age at diagnosis or match, years**	Median (range)	5.80	(1.72—9.60)	5.75	(1.67—9.63)
**Sex**	Female intact	7	(14.3%)	14	(14.3%)
	Female spayed	16	(32.7%)	32	(32.7%)
	Male intact	9	(18.4%)	18	(18.4%)
	Male neutered	17	(34.7%)	34	(34.7%)
**Age at gonadectomy, years**^**a**^	Median (range)	1.06	(0.22—4.64)	0.93	(0.21—4.71)
**Number of times moved**	0	40	(81.6%)	71	(72.4%)
	1	7	(14.2%)	23	(23.5%)
	2	2	( 4.1%)	2	( 2.0%)
	3	0	( 0.0%)	2	( 2.0%)
**Years lived at longest residence**	Median (range)	5	(1—8)	6	(2—9)
**Currently lives at longest residence?**	Yes	45	(91.8%)	88	(89.9%)
**Urbanicity of longest residence**	Rural	13	(26.5%)	25	(25.5%)
	Suburban	30	(61.2%)	66	(67.3%)
	Urban	6	(12.2%)	7	( 7.1%)
**Geographic region of longest residence**^**b**^	Pacific	3	( 6.1%)	13	(13.3%)
	Mountain	6	(12.2%)	12	(12.2%)
	Midwest	16	(32.7%)	24	(24.5%)
	Northeast	10	(20.4%)	23	(23.5%)
	South	14	(28.6%)	26	(26.5%)

^a^among gonadectomized dogs only

^b^Pacific: CA, OR, WA; Mountain: CO, AZ, NV, ID, UT, WY, MT, NM; Midwest: ND, SD, NE, KS, MN, IA, MO, WI, IL, IN, MI, OH; Northeast: PA, NJ, NY, CT, RI, MA; South: OK, TX, MD, DE, AR, LA, MS, AL, GA, FL, KY, TN, SC, NC, WV, VA

Most cases had multicentric lymphoma (*n* = 37; 70%), with remaining cases having gastrointestinal lymphoma (*n* = 8; 16%) or lymphoma primarily affecting one organ system (*n* = 4; 8%; included lingual, prostate, renal, and cardiac lymphoma). Eighty-six percent of cases (*n* = 42) were immunophenotyped using a combination of flow cytometry (*n* = 21), immunohistochemistry (*n* = 19), or PARR (*n* = 8). Cases were evenly divided between B-cell (*n* = 19) and T-cell (*n* = 20) subtypes. Three dogs with immunophenotyping were classified as “other” due to known differences in tumor biologic behavior: T-zone, marginal zone, and null-type.

### Household proximity to environmental pollutant sources

No proximities to environmental pollutant sources reached statistical significance in univariable analysis (Table 3). Four environmental pollutant sources (landfills, coal plants, high-voltage transmission lines, and nuclear power plants) had an overall exposure percentage < 5% and were thus excluded from individual statistical analysis. These variables were still included in the overall exposure score. For both cases and controls, the exposure score ranged from zero to five pollution sources, with a median of one pollution source. Having an exposure score of three or more was not statistically significantly associated with lymphoma risk (OR = 1.62, 95%CI 0.74–3.55).

**Table 3:** Univariable conditional logistic regression results for lymphoma cases versus matched controls

		**Cases** **(*n* = 49)**		**Controls (*n* = 98)**		**OR**^**c**^	**(95%CI)**
		n	(%)	n	(%)		
**Chemical plant**		10	(20.4%)	15	(15.3%)	1.39	(0.59—3.29)
**Municipal dump**		10	(20.4%)	20	(20.4%)	1.00	(0.41—2.43)
**Manufacturing plant**^a^		11	(22.4%)	15	(15.3%)	1.59	(0.67—3.75)
**Incineration plant**		17	(34.7%)	22	(22.4%)	1.83	(0.86—3.90)
**Railroad embankment track**		27	(55.1%)	51	(52.0%)	1.13	(0.57—2.26)
**Landfill**		3	( 6.1%)	4	( 4.1%)	–	–
**Coal plant**		0	( 0.0%)	1	( 1.0%)	–	–
**High-voltage transmission line**		1	( 2.0%)	2	( 2.0%)	–	–
**Nuclear power plant**		2	( 4.1%)	2	( 2.0%)	–	–
**Exposure index (continuous)**^**b**^		1	(0—5)	1	(0—5)	1.18	(0.92—1.51)
**3 + exposures (y/n)**		15	(30.6%)	21	(21.4%)	1.62	(0.74—3.55)
**Average ozone**	** > 70 ppb**	12	(24.5%)	35	(35.7%)	0.55	(0.24—1.26)
	**missing**	10	(20.4%)	19	(19.4%)	0.86	(0.36—2.05)
**Average PM2.5 (ug/m**^**3**^**)**^**b**^		8.2	(5.8—14.0)	8.4	(5.7—13.8)	1.04	(0.81—1.33)
**Secondhand smoke exposure (y/n)**		6	(12.2%)	12	(12.2%)	1.00	(0.35—2.83)

^a^Manufacturing plant includes rubber, leather or textile manufacturing plants

^b^median (range) shown

^c^Odds ratios (ORs) and 95% confidence intervals (CIs) are shown

**Fig. 1:**
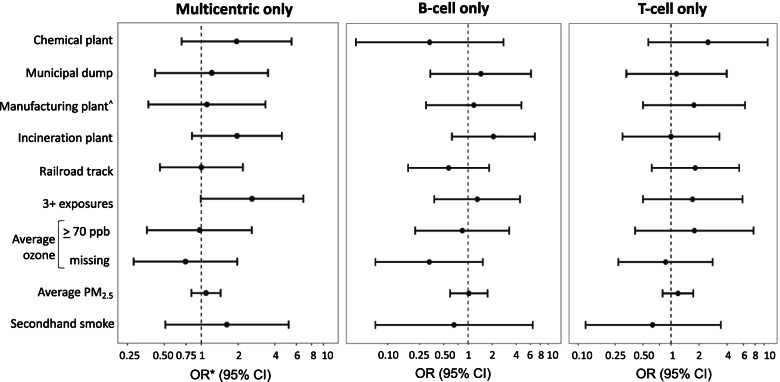
Univariable conditional logistic regression analysis results for lymphoma case subgroups versus matched controls. *Odds ratios (ORs) and 95% confidence intervals (CIs) are shown in log scale. ^^^Manufacturing plant includes rubber, leather or textile manufacturing plants

When limiting analyses to 37 cases of multicentric lymphoma and their matched controls, results for individual exposure sources and lymphoma risk generally mirrored the primary analysis (Fig. 1, Additional File 1). However, having an exposure score of three or more was associated with over double the lymphoma risk; this association approached statistical significance (OR = 2.60, 95%CI 0.99–6.86; *p*-value 0.053).

Sample size was limited when evaluating B-cell lymphoma (*n* = 19) separately from T-cell lymphoma (*n* = 20). Some differences in trends emerged, although no associations achieved statistical significance (Fig. 1, Additional File 1). For example, exposure to railroad embankment tracks appeared lower among B-cell lymphoma cases (OR = 0.58, 95%CI 0.18–1.83) and higher among T-cell lymphoma cases OR = 1.82, 95%CI 0.62–5.40).

### Annual county average ozone and PM_2.5_ levels

Average annual county-level ozone and PM_2.5_ levels were unavailable for 29 and 43 dogs, respectively. Although not statistically significant, having an average annual county-level ozone ≥ 70 ppb appeared to be less common among dogs with lymphoma (OR = 0.55, 95%CI 0.24 – 1.26; Table 3). No statistically significant associations were observed in the subtype analyses. No statistically significant associations between PM_2.5_ levels and lymphoma were noted in overall or subtype analyses.

### Secondhand smoke exposure

Only six households from the case population and twelve households from the control population reported any secondhand smoke exposure. There was no statistically significant difference between household secondhand smoke exposure and lymphoma in overall or subtype analyses (Table 3).

## Discussion

Golden Retrievers have an increased lymphoma risk in the United States, likely due to both environmental and genetic risk factors. This study investigated the relationship between lymphoma risk and household proximity to potential environmental pollutant sources, county levels of air pollution measured as ozone and PM_2.5_ levels, or owner-reported secondhand smoke exposure in a nationwide cohort of Golden Retrievers. Using a nested case–control study of 49 lymphoma cases and 98 age- and sex-matched controls, we found no statistically significant associations between proximity to environmental pollutant exposure sources, markers of air pollution at the county level, or reported secondhand smoke exposure and lymphoma.

We found no statistically significant associations between proximity to chemical plants, municipal dumps, manufacturing plants, incineration plants, or railroad embankment tracks and overall lymphoma risk. Due to a low number of dogs (< 5%) living near landfills, coal plants, high-voltage transmission lines, or nuclear power plants, we were unable to statistically evaluate whether these pollutant sources were individually associated with lymphoma risk. Prior research has indicated exposure to various environmental pollutants may increase risk of NHL in people [7, 29–32]. The lack of statistically significant associations between individual manufacturing sites and chemical suppliers in this population may be due to limitations in sample size or distinct etiologies for different lymphoma subtypes, as hypothesized in human populations [33].

Consistent with prior reports for this breed, the majority of cases were multicentric lymphoma (70%) and there was an even distribution of B- (45%) and T-cell (48%) subtypes among cases that were immunophenotyped (86%) [2]. When comparing B- and T-cell lymphoma, a few differences were observed. Household proximity to incineration plants appeared more common among B-cell lymphoma cases, whereas proximity to railroad embankment tracks appeared more common among T-cell lymphoma cases. However, neither of these differences reached statistical significance. Proximity to incineration plants and chemical manufacturers were previously reported to increase lymphoma risk in Boxer dogs, which most commonly develop T-cell lymphoma [2, 3, 6]. Since previous literature on specific environmental exposures did not differentiate lymphoma subtypes, it is difficult to determine whether our findings are comparable. It is possible that the differences in the lifestyles and exposure patterns of Golden Retrievers and Boxers, background genetic susceptibility, or breed tendency toward specific lymphoma subtypes played a role in our findings. Although environmental exposure patterns reported here did not reach formal statistical significance, this work supports further research into differing environmental exposure risk factors between B- and T-cell lymphoma subtypes.

Ecologic studies on human lymphoma risk have reported mixed results with respect to residential proximity to different sources of carcinogen emissions. One case–control study in the United States showed no statistically significant associations between overall or subtype-specific NHL risk and proximity to solid waste incinerators or quartile of facility-specific dioxin emissions within 3 or 5 km of the residence [34]. In contrast, a case–control study from France reported an increased risk of NHL among those living in census blocks near solid waste incinerators [35]. Furthermore, increased relative risk for NHL overall was seen among people residing within a half-mile to 2-mile radius of stone, glass or clay factories in Iowa and Minnesota [36].

When examining cumulative exposure burden across environmental pollution sources and lymphoma prevalence, we saw dogs with three or more exposures had a non-statistically significant increased risk of multicentric lymphoma. This may suggest individually insignificant exposures produce a synergistic effect with other exposure sites on lymphoma risk, either through accumulated exposure of one or two key pollutants from multiple sites or from different environmental compounds combining their mutagenic effects. Living near multiple potential environmental pollutant sites may also serve as an indicator of a more urban residence, and thereby higher exposure to other air, water, or soil-borne contaminants. It may also indicate greater socioeconomic household disadvantage, and reduced access to care [37, 38].

Household socioeconomic status was not a variable accounted for within this study but warrants further research, as this has been shown to interact with environmental emissions and cumulative cancer burden in human populations [39].

Unlike previous studies in Boxer dogs, living in a county with elevated average ozone levels (over 70 ppm) was not associated with lymphoma risk in this population [40]. This was unexpected, as increased near-surface ozone levels generally indicate higher industrial pollutant burden. There are a few possible explanations for this finding. First, 29 dogs were missing ozone data in our population; this may influence our results, as ozone was more likely to be missing for dogs residing in rural areas (*n* = 16 [42%] rural dogs, *n* = 12 [13%] suburban dogs, *n* = 1 [8%] urban dogs). Second, we assessed summary ozone and PM_2.5_ data, which may be missing key ecological events such as wildfires that cause spikes in atmospheric pollution or overlooking the key etiological window for those events. Finally, other exposures more commonly found in rural areas, such as herbicides or nitrates in the water, may play a greater role in lymphoma risk than atmospheric pollution [6, 8, 41]. In human studies, air quality was reported to be improved in more rural locations, but markers of water pollution were more severe [42].

No association between secondhand smoke exposure and lymphoma was found in our study. Only 12% of dogs in our study had any reported secondhand smoke exposure; among them, the median reported exposure was one hour per day. It is possible that exposure to secondhand smoke was too uncommon in this population to detect a modest effect, or that exposures were under-reported. Smoking has been linked to follicular lymphoma in people [25], but studies are conflicting [43].

The use of geocoding in this study carries limitations. Because Google Maps is updated every two years, the accuracy of our searches was limited to the structures present within the last two years from the date of the search. As such, pollutant sources may have emerged or been remediated during the etiologic window of interest. Additionally, proximity to pollutant sources is an indirect assessment of exposure to pollutants and, while economically more feasible to study, is not as robust as evaluating individual exposures via biomarker measurements. There are also inherent limitations to an ecologic study design in that we are attributing population-level exposures to an individual-level disease risk. Many individual factors may influence the accuracy of our associations. For instance, the amount of time a dog spends outdoors will affect its exposure to ambient air pollution. May household dogs spend most of their time indoors, and we are less able to assess indoor air pollution with an ecologic study design. We were also underpowered to assess interactions with environmental exposures and diet, obesity, or genetic pathways of chemical detoxification.

Due to the young age of the cohort at the time of this study, sample size was limited in both overall and lymphoma subtype analyses. While we aimed to assess subtype-specific risk factors for lymphoma, we were only able to compare multicentric, B- and T-cell lymphoma subtypes and had limited sample size for those comparisons. While the B-cell lymphoma cases in our study are predominately diffuse large B-cell lymphoma and the T-cell lymphoma cases are predominately peripheral T cell lymphoma not otherwise specified, these are still heterogeneous categories. Some lymphoma subtypes may have a stronger genetic etiologic component (e.g., T zone lymphoma/leukemia, which is common among Golden Retrievers), whereas other subtypes may have a stronger environmental etiologic component (e.g., diffuse large B-cell lymphoma). As the GRLS population continues to age, we may see an expansion in certain lymphoma subtypes, and shift in subtype prevalence, thereby acquiring the power level to conduct subgroup analyses.

Despite these limitations, this study had several key strengths. All lymphoma cases were pathologist- or laboratory-confirmed, with subtyping available for most cases. Control dogs all underwent annual physical examinations and diagnostic testing, increasing our confidence that they were lymphoma-free at the time of matching. Cases and controls were also matched on age, sex, and timing of gonadectomy to decrease potential confounding [44–46]. Importantly, the prospective longitudinal design of the cohort allowed us to account for mobility of study participants and duration of residence when evaluating environmental exposures.

In conclusion, in this nested case–control study of lymphoma risk in Golden Retrievers, we did not detect any statistically significant associations between proximity to environmental pollutant sources, average annual ozone or PM_2.5_ levels, or reported secondhand smoke exposure and lymphoma risk in this breed. However, dogs exposed to multiple environmental pollutant sources had a non-significantly elevated lymphoma risk. Additionally, we identified some potential differences in risk patterns when comparing B- and T-cell lymphoma. Further research is required to assess the significance of exposure to environmental pollutant sources and air quality in lymphoma risk among dogs, and whether this risk may differ by lymphoma subtype or breed. It is important to consider cumulative exposure burden as well as individual geographic pollutant exposure sources. As the current GRLS cohort ages, further studies will be able to re-assess the impact of the environmental exposure sources explored here, in addition to biomarkers for exposure and questionnaire data for more robust assessment of true exposure. Regional subgroup analyses for exposure risk coefficients should also be considered if sample size allows.

## Supplementary Information

**Additional file 1.** Univariable conditional logistic regression analysis results for lymphoma case subgroups versus matched controls. Description: Exposure frequencies, odds ratios (ORs) and 95% confidence intervals (CIs) for lymphoma case subgroup analyses.

## Data Availability

The datasets used and/or analyzed during the current study are available from the corresponding author on reasonable request.

## Abbreviations

NHL: Non-Hodgkin’s lymphoma
GRLS: Golden Retriever Lifetime Study
PARR: Polymerase Chain Reaction for Antigen Receptor Rearrangement
OR: Odds Ratio
CI: Confidence Interval
EPA: Environmental Protection Agency
PM_2.5_: Particulate matter 2.5 microns or less
