# Supplementary material for: Environmental exposures and lymphoma risk: a nested case–control study using the Golden Retriever Lifetime Study cohort
Source: Canine Med Genet. 2022 Jul 15;9:10. doi: 10.1186/s40575-022-00122-9 (PMC9287967; doi:10.1186/s40575-022-00122-9)
Supplement: Supplementary file 1 — Additional file 1. Univariable conditional logistic regression analysis results for lymphoma case subgroups versus matched controls. Description: Exposure frequencies, odds ratios (ORs) and 95% confidence intervals (CIs) for lymphoma case subgroup analyses. [file 40575_2022_122_MOESM1_ESM.pdf]

	MULTICENTRIC LYMPHOMA ONLY				B-CELL LYMPHOMA ONLY				T-CELL LYMPHOMA ONLY			
	Cases (n=37) n (%)	Controls (n=74) n (%)	OR*	(95%CI)	Cases (n=19) n (%)	Controls (n=38) n (%)	OR	(95%CI)	Cases (n=20) n (%)	Controls (n=40) n (%)	OR	(95%CI)
**Chemical plant**	8 (21.6%)	9 (12.2%)	1.95	(0.69 - 5.48)	1 (5.3%)	6 (15.8%)	0.33	(0.04 - 2.77)	5 (25.0%)	5 (12.5%)	2.50	(0.57 - 10.96)
**Municipal dump**	8 (21.6%)	14 (18.9%)	1.22	(0.42 - 3.52)	4 (21.1%)	6 (15.8%)	1.44	(0.34 - 6.11)	5 (25.0%)	9 (22.5%)	1.15	(0.33 - 3.97)
**Manufacturing plant^**	6 (16.2%)	11 (14.9%)	1.11	(0.37 - 3.35)	4 (21.1%)	7 (18.4%)	1.18	(0.30 - 4.63)	5 (25.0%)	6 (15.0%)	1.77	(0.50 - 6.24)
**Incineration plant**	15 (40.5%)	19 (25.7%)	1.96	(0.84 - 4.57)	7 (36.8%)	8 (21.1%)	2.08	(0.63 - 6.81)	5 (25.0%)	10 (25.0%)	1.00	(0.30 - 3.32)
**Railroad embankment track**	20 (54.1%)	40 (54.1%)	1.00	(0.46 - 2.19)	9 (47.4%)	23 (60.5%)	0.58	(0.18 - 1.83)	12 (60.0%)	18 (45.0%)	1.82	(0.62 - 5.40)
**Exposure index (continuous)#**	1 (0 - 5)	1 (0 - 5)	1.23	(0.92 - 1.66)	1 (0 - 5)	1 (0 - 4)	1.05	(0.70 - 1.57)	1 (0 - 5)	1 (0 - 5)	1.19	(0.81 - 1.74)
**3+ exposures (y/n)**	12 (32.4%)	12 (16.2%)	2.60	(0.99 - 6.86)	6 (31.6%)	10 (26.3%)	1.31	(0.38 - 4.45)	6 (30.0%)	8 (20.0%)	1.70	(0.50 - 5.87)
**Average ozone ≥ 70 ppb**	10 (27.0%)	24 (32.4%)	0.74	(0.28 - 1.97)	3 (15.8%)	13 (34.2%)	0.33	(0.07 - 1.53)	7 (35.0%)	17 (42.5%)	0.88	(0.27 - 2.81)
**missing**	8 (21.6%)	15 (20.3%)	0.97	(0.36 - 2.59)	5 (26.3%)	8 (21.1%)	0.85	(0.22 - 3.26)	5 (25.0%)	6 (15.0%)	1.79	(0.41 - 7.72)
**Average PM2.5 (continuous)#**	8 (5.8 - 14.0)	9 (5.7 - 13.8)	1.09	(0.83 - 1.44)	8 (5.8 - 10.6)	8 (5.9 - 13.8)	1.02	(0.60 - 1.75)	9 (6.0 - 11.4)	9 (5.7 - 12.8)	1.19	(0.81 - 1.74)
**Secondhand smoke exposure**	6 (16.2%)	8 (10.8%)	1.62	(0.51 - 5.19)	1 (5.3%)	3 (7.9%)	0.67	(0.07 - 6.41)	2 (10.0%)	6 (15.0%)	0.63	(0.12 - 3.44)

^manufacturing plant includes rubber, leather or textile manufacturing plants

#median (range) shown

*Exposure frequencies, odds ratios (ORs) and 95% confidence intervals (CIs) are shown
